# *Pseudomonas aeruginosa* serotypes in nosocomial pneumonia: prevalence and clinical outcomes

**DOI:** 10.1186/cc13697

**Published:** 2014-01-15

**Authors:** Qin Lu, Philippe Eggimann, Charles-Edouard Luyt, Michel Wolff, Michael Tamm, Bruno François, Emmanuelle Mercier, Jorge Garbino, Pierre-François Laterre, Holger Koch, Verena Gafner, Michael P Rudolf, Erkan Mus, Antonio Perez, Hedvika Lazar, Jean Chastre, Jean-Jacques Rouby

**Affiliations:** Multidisciplinary Intensive Care Unit, Department of Anesthesiology and Critical Care Medicine, La Pitié-Salpêtrière Hospital, Assistance Publique Hôpitaux de Paris, UPMC Paris 6, 47-83 boulevard de l’Hôpital, Paris, 75013 France; Department of Adult Intensive Care Medicine, Centre Hospitalier Universitaire Vaudois, Rue du Bugnon 46, 1011 Lausanne, Switzerland; Medical Intensive Care Unit, La Pitié-Salpêtrière Hospital, Assistance Publique Hôpitaux de Paris, UPMC Paris 6, 47-83 boulevard de l’Hôpital, 75013 Paris, France; Reanimation Medicale et Infectieuse, Hopital Bichat-Claude Bernard, 46 Rue Henri Huchard, 75018 Paris, France; Pneumonia Clinic, University Hospital Basel, Petersgraben 4, 4031 Basel, Switzerland; Intensive Care Unit, CIC-P 0801 Inserm, CHU Dupuytren, 2 avenue Martin-Luther-King, 87042 Limoges, France; Réanimation médicale, Hôpital Bretonneau, 2 Boulevard Tonnellé, 37000 Tours, France; University Hospitals of Geneva, Rue Gabrielle-Perret-Gentil 4, 1205 Geneva, Switzerland; Intensive Care Unit, St Luc University Hospital, UCL, Avenue Hippocrate 10, 1200 Woluwe-Saint-Lambert, Belgium; Kenta Biotech, Wagistrasse 25, CH-8952 Zürich-Schlieren, Switzerland

## Abstract

**Introduction:**

*Pseudomonas aeruginosa* frequently causes nosocomial pneumonia and is associated with poor outcome. The purpose of this study was to assess the prevalence and clinical outcome of nosocomial pneumonia caused by serotype-specific *P. aeruginosa* in critically ill patients under appropriate antimicrobial therapy management.

**Methods:**

A retrospective, non-interventional epidemiological multicenter cohort study involving 143 patients with confirmed nosocomial pneumonia caused by *P. aeruginosa*. Patients were analyzed for a period of 30 days from time of nosocomial pneumonia onset. Fourteen patients fulfilling the same criteria from a phase IIa studyconducted at the same time/centers were included in the prevalence calculations but not in the clinical outcome analysis.

**Results:**

The prevalence of serotypes was: O6 (29%), O11 (23%), O10 (10%), O2 (9%), and O1 (8%). Serotypes with a prevalence of less than 5% were found in 13% of patients, 8% were classified as not typeable. Across all serotypes, 19% mortality, 70% clinical resolution, 11% clinical continuation, and 5% clinical recurrence were recorded. Age and higher APACHE II (Acute Physiology and Chronic Health Evaluation II) were predictive risk factors associated with probability of death and lower clinical resolution for *P. aeruginosa* nosocomial pneumonia. Mortality tends to be higher with O1 (40%) and lower with O2 (0%); clinical resolution tends to be better with O2 (82%) compared to other serotypes. Persisting pneumonia with O6 and O11 was, respectively, 8% and 21%; clinical resolution with O6 and O11 was, respectively, 75% and 57%.

**Conclusions:**

In *P. aeruginosa* nosocomial pneumonia, the most prevalent serotypes were O6 and O11. Further studies including larger group sizes are needed to correlate clinical outcome with virulence factors of *P. aeruginosa* in patients with nosocomial pneumonia caused by various serotypes; and to compare O6 and O11, the two serotypes most frequently encountered in critically ill patients.

## Introduction

*Pseudomonas aeruginosa* is the pathogen responsible for approximately 20% of ventilator-associated pneumonia (VAP) and is one of the most difficult pathogens to treat [1, 2]. VAP caused by *P. aeruginosa* has the poorest outcome of all intensive care unit (ICU) infections. Overall mortality due to *P. aeruginosa* has been shown to be as high as 70% [3, 4], and directly attributable mortality rates are approximately 40% [3, 5].

Lipopolysaccharide (LPS) is an important virulence factor in *P. aeruginosa*, exerting direct endotoxic effects. The tripartite nature of LPS divides the molecule into a hydrophobic lipid A region, a central core oligosaccharide region, and a repeating polysaccharide portion referred to as O antigen or O polysaccharide [6, 7]. The most complete serotyping system for *P. aeruginosa*, the International Antigenic Scheme (IATS), consists of 20 standard O serotypes [8, 9]. Current epidemiology data indicate that, of these 20 serotypes, IATS-O1, serogroup 2 (IATS-O2, IATS-O5, and IATS-16), IATS-O6, and IATS-O11 are responsible for 70% of *P. aeruginosa* infections [10, 11].

The relationship between the virulence of *P. aeruginosa* and different serotypes has been studied [12–14]. Strains exhibiting exotoxin U (ExoU), one of the toxins secreted by the type III secretion system (TTSS), were frequently serotyped as O11, whereas serotype O6 strains were associated with a negative ExoU phenotype [12, 15]. In an experimental model of pneumonia, serotype O11 was found to be associated with increased lung injury [15]. Moreover, it has been reported that some serotypes are able to induce high resistance of *P. aeruginosa* to antibiotics [16–18].

Little is known, however, about the prevalence of *P. aeruginosa* serotypes in nosocomial pneumonia and the correlation between serotypes and clinical outcome. The primary objective of the study was to assess the prevalence of *P. aeruginosa* serotypes in critically ill patients with nosocomial pneumonia. The secondary objective was to report clinical outcome of nosocomial pneumonia caused by different serotypes of *P. aeruginosa*.

## Materials and methods

### Study design and patients

This was a retrospective, multicenter, and non-interventional epidemiological cohort study conducted between 2007 and 2009 in the ICU of nine hospitals in France, Switzerland, and Belgium. Patients with confirmed hospital-acquired pneumonia (HAP) and VAP caused by *P. aeruginosa* were analyzed for a period of 30 days from the time of diagnosis of pneumonia. The following ethics committees approved the study: Basel Ethikkommission beider Basel EKBB, Geneva Commission d’Éthique du Département de Médecine, Hôpitaux Universitaires de Gèneve, Lausanne Commission d’Éthique de la Recherche Clinique de la Faculté de Biologie et Médecine, le Comité de Protection des Personnes, Ile de France-VI, Brussels Université Catholique de Louvain, Faculté de Médecine, Commission d’Éthique Biomédicale Hospitalo-Facultaire. Because it was a retrospective epidemiological study, no informed consent was needed. Written informed consent was obtained in only 14 patients with serotype O11 HAP or VAP when they were included in a phase IIa study [19].

### Inclusion criteria

Included patients were at least 18 years old, required intensive care management with HAP and VAP, were expected to survive longer than 72 hours, and fulfilled one of the following two criteria [20]:

1. Confirmed microbiological diagnosis of HAP and VAP caused by *P. aeruginosa* isolated from lower respiratory tract specimen with significant threshold of at least 1 × 10^4^ colony-forming units (CFU)/mL for bronchoalveolar lavage (BAL) [21] or 1 × 10^3^ CFU/mL for protected mini-lavage (mini-BAL) [22], or below if under treatment with antibiotics, and presence of a new or progressing pulmonary infiltrate, plus one of the following three criteria: (a) fever of greater than 38°C, (b) white blood cell count of greater than 10,000/mm^3^, or (c) purulent sputum

Or

2. Confirmed microbiological diagnosis of HAP and VAP caused by *P. aeruginosa* isolated from endotracheal aspirate (ETA) with at least 1 × 10^6^ CFU/mL and a modified clinical pulmonary infection score (MCPIS) of higher than 6 points [23].

Patients with nosocomial pneumonia caused by multiple bacteria were included in the study. Multiple bacteria were defined as more than one pathogen reaching significant threshold isolated from lower respiratory tract specimen.

### Exclusion criteria

During the same period of time and at the same centers, a phase IIa study which included 14 patients with O11 HAP or VAP was performed [19]. To reflect the correct prevalence of O11 serotype during the observation period, these patients were included in the prevalence calculation. They were, however, excluded from the clinical outcome analysis as they were treated with a combination of standard antibiotic treatment plus an adjunctive immunotherapy [19]. Exclusion criteria were applicable to only the 14 patients who participated in the phase IIa study. They included use of any investigational drug within 30 days prior to study commencement or during the study; patients with a known complement deficiency associated with systemic lupus erythematosus, paroxysmal nocturnal hemoglobinuria, hereditary angioedema, membranoproliferative glomerulonephritis, collagen vascular disease, autoimmune hepatitis, primary biliary cirrhosis, scleroderma, or recurrent *Neisserial* infections; or patients with confirmed HIV infection. Transplant patients and/or patients treated with systemic immuno-suppressive drugs (except prednisone or prednisolone), patients with a known liver function deficiency, and neutropenic patients (absolute neutrophil count of less than 1,000 cells/μL) were also excluded [19].

### Assessments

Serotypes of *P. aeruginosa* isolated from lower respiratory tract specimens were determined by agglutination using serotype-specific antisera (Bio-Rad, Marnes-la Coquette, France) in accordance with the instructions of the manufacturer.

Patient clinical characteristics, Acute Physiology and Chronic Health Evaluation II (APACHE II) score [24], sequential organ failure assessment (SOFA) score, MCPIS, risk factors, microbiological assessments (including serotyping) in ETA or BAL/mini-BAL, blood culture, chest radiographies, and laboratory parameters were assessed at inclusion. Scores, laboratory parameters, chest radiographies, and body temperature were collected as frequently as possible from day 1 to day 15, at days 21 and 30, or end of study (EOS) until patient died or was lost for follow-up.

Data collected at day 30 or EOS, whichever date was earlier, included clinical outcome, scores, laboratory parameters, chest radiographies, body temperature, discharge from the ICU, and discharge from the hospital.

Clinical outcome was defined as follows:

- ● Continuation: clinical signs and symptoms of pneumonia present during the whole assessment period until day 30 (or EOS)
- ● Resolution: complete resolution of pneumonia signs and symptoms present at the time of enrollment, no new symptoms or complications attributable to the pneumonia, no recurrence of pneumonia, and alive, until day 30 (or EOS)
- ● Recurrence: return of all clinical signs and symptoms of HAP/VAP, including infiltrates after initial clinical resolution
- ● Death: assessment period until day 30 (or EOS). Actual mortality was compared with predicted mortality according to APACHE II score [24].

Microbiological outcome was defined as follows:

- ● Continuation: positive culture of *P. aeruginosa* in ETA or (mini)-BAL over the whole assessment period until day 30 (or E OS) associated with persisting clinical signs and symptoms of pneumonia
- ● Resolution: baseline isolate not present in repeated culture from original infection site
- ● Recurrence: isolation of *P. aeruginosa* from a culture taken after the resolution of pneumonia
- ● Colonization: positive culture of *P. aeruginosa* in ETA or (mini)-BAL over the whole assessment period until day 30 (or EOS) without any clinical pneumonia signs and symptoms.

### Statistical analysis

Descriptive statistics were used for patient characteristics and outcome variables. Categorical variables were expressed as percentage, and continuous variables as mean ± standard deviation or as median and 25% to 75% interquartile range according to data distribution. Clinical and microbiological outcomes were considered binary endpoints and these were analyzed by using Bayesian logistic regression models. Logistic regression was used for binary outcomes and model based *P* values, using likelihood ratio tests. Time-to-event endpoints (survival and clinical and microbiological resolution) were analyzed by using Kaplan-Meier and Cox proportional hazards regression models. Analysis was done without adjustment for risk factors, with adjustment for different serotypes, and with adjustment for risk factors (age, APACHE II score, CPIS, SOFA score, and adequacy of antibiotic treatment). Analyses of data were performed by using R 2.10.1 and WinBUGS 1.4.3.

## Results

### Patient demographics and characteristics

**Figure 1:**
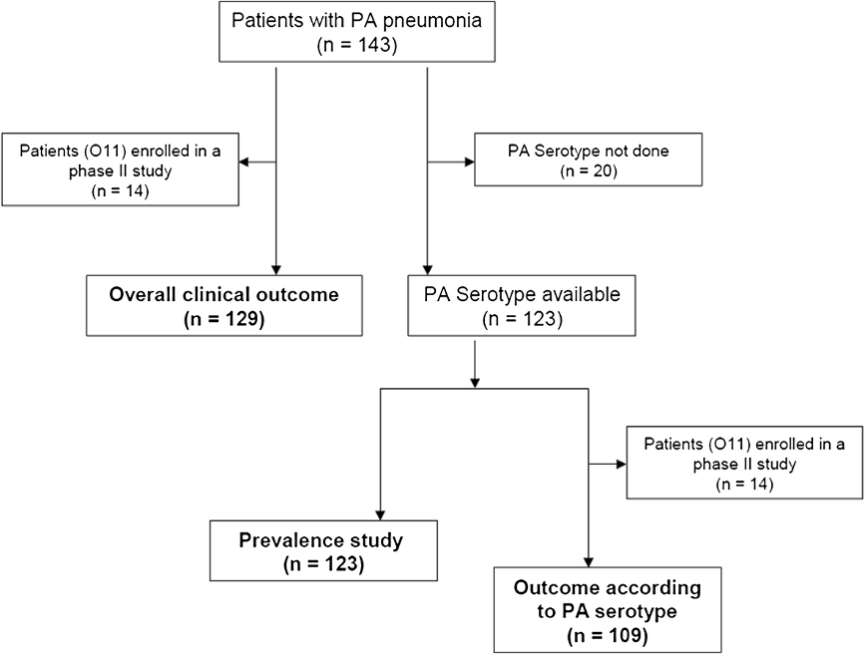
**Patients’ flow chart.** O11, serotype O11; PA, *Pseudomonas aeruginosa*.

Patients’ flow chart is shown in Figure 1. Of 143 patients, 123 patients were included in the prevalence analysis and 129 in the overall clinical outcome analysis. Among 123 patients with serotype available, 114 patients had VAP (93%) and 9 had HAP (7%). Clinical and demographic data are shown in Table 1. At pneumonia onset, median length of hospital stay was 13 days, median length of ICU stay was 8 days, and median duration of mechanical ventilation was 8 days.

**Table 1:** **Clinical characteristics of patients with nosocomial pneumonia caused by different** ***Pseudomonas aerugionsa*** **serotypes (n = 123)**

Serotypes	All	O1	O2	O6	O10	O11	PL5	NT
**VAP/NP, n (%)**	**114/123 (93)**	9/10 (90)	10/11 (91)	35/36 (92)	10/12 (83)	26/28 (93)	15/16 (94)	9/10 (90)
**Age, years**	**61 (43 to 72)**	65 (56 to 74)	63 (58 to 70)	57 (39 to 67)	59 (40 to 77)	65 (42 to 78)	64 (47 to 71)	64 (59 to 72)
**Male%**	**75**	40	73	81	92	68	81	80
**Antibiotics at pneumonia onset, n, %**	**84 (68)**	5 (50)	8 (73)	28 (78)	12 (100)	13 (46)	12 (75)	6 (60)
**Inappropriately treated %**	**11**	0	0	14	8	14	6	0
**MCPIS**	**7.0 (5.0 to 9.0)**	8.0 (5.8 to 9.5)	9.0 7 to 10)	7 (5 to 8)	6 (4 to 8)	8.5 (7 to 9.5)	7.5 5 to 9)	6.0 (4 to 8)
**APACHE II score**	**17 (12 to 22)**	17 (11 to 29)	15 (11 to 17)	17 (11to 23)	17 (11 to 29)	17 (14 to 20)	19 (12 to 26)	16 (12 to 19)
**SOFA score**	**7.0 (5.0 to10.0)**	9.5 (6.5 to 15.0)	5 (4 to 7)	7 (5 to 10)	9.5 (6.5 to 15.0)	6.0 (4.5 to 8.0)	7 (6.0 to 9.0)	6.0 (4.0 to 9.2)
**Hospital length of stay at inclusion, days**	**13 (7 to 14)**	14 (8 to 45)	8 (4 to 18)	12 (7 to 25)	13 (8 to 19)	18 (8 to 34)	12 (6 to 24)	14 (8 to 25)
**ICU length of stay at inclusion, days**	**8 (4 to 17)**	11 (4 to 17)	5 (3 to 12)	7 (5 to 19)	8 (3 to 13)	9 (6 to 19)	8 (4 to 15)	9 (5 to 14)
**Duration of mechanical ventilation at inclusion, days**	**8 (5 to 17)**	10.5 (7 to 17)	5 (3 to 12)	8 (5 to18)	9 (4 to 13)	9 (5 to 17)	8 (4 to 15)	9 (6 to 14)

Data are expressed as median and 25%-75% interquartile. Nosocomial pneumonia (NP) includes hospital-acquired pneumonia and ventilator-associated pneumonia (VAP). APACHE II, Acute Physiology and Chronic Health Evaluation II; ICU, intensive care unit; MCPIS, modified clinical pulmonary infection score; NT, not typeable; PL5, sum of all serotypes with a prevalence of less than 5%; SOFA, sequential organ failure assessment.

### Prevalence of *Pseudomonas aeruginosa*serotypes

The rates of prevalence of serotypes were, respectively, O6 (29%), O11 (23%), O10 (10%), O2 (9%), and O1 (8%). The sum of all serotypes with a prevalence of less than 5% (PL5) was 13%, and 8% of all patients had a *P. aeruginosa* infection classified as not typeable (NT) (Table 2).

**Table 2:** ***Pseudomonas aeruginosa*** **serotype distribution (n = 123)**

Serotype	Number	Prevalence, percentage
**O6**	**36**	**29.3**
**O11**	**28**	**22.8**
**O10**	**12**	**9.8**
**O2**	**11**	**8.9**
**O1**	**10**	**8.1**
**NT**	**10**	**8.1**
O3	5	4.1
O4	3	2.4
O7	3	2.4
O9	2	1.6
O8	1	0.8
O12	1	0.8
O15	1	0.8

Serotypes with a prevalence of more than 5% are expressed in boldface. NT, not typeable.

**Figure 2:**
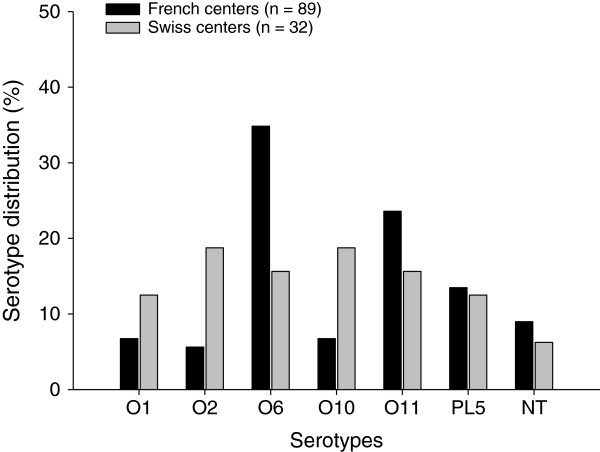
***Pseudomonas aeruginosa*** **serotype distribution in French (black bars) and Swiss (grey bars) investigator centers.** n, number of patients.

The prevalence of *P. aeruginosa* serotypes in French and Swiss investigator centers is shown in Figure 2. The distribution of *P. aeroginosa* serotypes by each investigator site is shown in Table 3.

**Table 3:** ***Pseudomonas aeruginosa*** **serotype distribution by each investigator site**

Investigator site		O1	O2	O6	O10	O11	PL5	NT	Total
**1**	n	2	0	8	2	1	2	1	16
	**%**	**12.5%**	**0.0%**	**50.0%**	**12.5%**	**6.3%**	**12.5%**	**6.3%**	**100%**
**2**	n	1	0	3	2	2	3	2	13
	**%**	**7.7%**	**0.0%**	**23.1%**	**15.4%**	**15.4%**	**23.1%**	**15.4%**	**100%**
**3**	n	2	4	12	1	14	4	3	40
	**%**	**5.0%**	**10.0%**	**30.0%**	**2.5%**	**35.0%**	**10.0%**	**7.5%**	**100%**
**4**	n	0	0	3	1	3	1	2	10
	**%**	**0.0%**	**0.0%**	**30.0%**	**10.0%**	**30.0%**	**10.0%**	**20.0%**	**100%**
**5**	n	1	1	5	0	1	2	0	**10**
	**%**	**10.0%**	**10.0%**	**50.0%**	**0.0%**	**10.0%**	**20.0%**	**0.0%**	**100%**
**6**	n	4	5	4	1	2	2	0	**18**
	**%**	**22.2%**	**27.8%**	**22.2%**	**5.6%**	**11.1%**	**11.1%**	**0.0%**	**100%**
**7**	n	0	1	1	5	1	2	2	**12**
	%	**0.0%**	**8.3%**	**8.3%**	**41.7%**	**8.3%**	**16.7%**	**16.7%**	**100%**

Investigator sites with more than 10 patients were analyzed. Percentages of serotype are expressed in boldface. NT, not typeable; PL5, sum of all serotypes with a prevalence of less than 5%.

### Overall clinical outcome

On day 30, across all serotypes, a 19% mortality, a 70% clinical resolution, an 11% clinical continuation, and a 5% clinical recurrence were recorded. Mortality was associated with age (*P* = 0.012) and high APACHE II score (*P* = 0.003). Clinical resolution was not associated with any of the risk factors—APACHE II score, CPIS score, SOFA score, adequate antibiotic treatment and age—or interaction of risk factors. Clinical continuation was associated with high APACHE II score (*P* <0.001).

Time to death was associated with age (*P* = 0.015) and high APACHE II score (*P* = 0.010). Time to event analysis of clinical resolution was associated with high APACHE II score (*P* = 0.008).

Among 102 patients with microbiological information available, a 41% microbiological resolution, a 25% microbiological continuation, a 23% colonization, and an 11% microbiological recurrence were recorded. Time to microbiological resolution was associated with APACHE II score (*P* <0.001) and higher MCPIS (*P* <0.007).

### Clinical outcome according to *Pseudomonas aeruginosa*serotype

**Figure 3:**
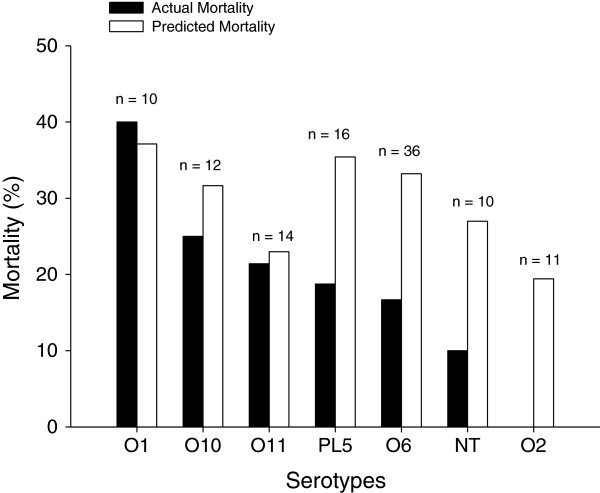
**Percentages of predicted and actual mortality of patients with nosocomial pneumonia caused by different** ***Pseudomonas aeruginosa*** **serotypes.** Black bars indicate actual mortality, and white bars indicate predicted mortality estimated according to Acute Physiology and Chronic Health Evaluation II (APACHE II) score. NT, not typeable; PL5, sum of all serotypes with a prevalence of less than 5%.

Among 109 patients (Figure 1), 15% of patients had nosocomial pneumonia caused by multiple bacteria, which were evenly distributed among the different serotype groups. An 18% mortality and a 69% clinical resolution were recorded. The highest mortality rate was observed in patients with serotype O1 infections (40%), and the lowest in patients with serotype O2 infections (0%) (Figure 3). Actual mortality and predicted mortality by APACHE II score were similar in patients infected by serotypes O1, O10, and O11, whereas actual mortality was lower than predicted mortality in patients infected by serotypes PL5, O6, NT, and O2 (Figure 3).

**Figure 4:**
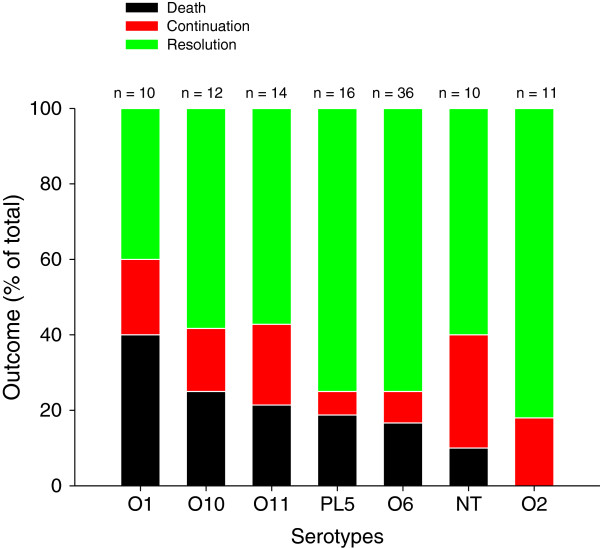
**Proportions of death (black bars), persisting pneumonia (or continuation) (red bars), and clinical resolution (green bars) of patients with nosocomial pneumonia caused by different** ***Pseudomonas aeruginosa*** **serotypes.** NT, not typeable; PL5, sum of all serotypes with a prevalence of less than 5%.

As shown in Figure 4, the highest clinical resolution rate was found in patients with serotype O2 (82%) and the lowest in patients with serotype O1 (40%). The serotypes O1, O10, O11, and NT had lower rates of clinical resolution (40%, 58%, 57%, and 60%) and higher rates of continuation (20%, 17%, 21%, and 30%), whereas the serotypes O6 and the PL5 group had continuation rates below 10%. The highest rates of recurrence were found in patients with serotype O1, whereas none of the 11 patients with serotype O10 and PL5 relapsed.

Mean time to death across all groups with nosocomial pneumonia was 11.4 days (Table 4). Overall time to resolution was 12.1 days, the shortest being for the patients with O2 infections (10 days) and longest being for the patients with O11 infections (13.4 days) (Table 4).

**Table 4:** **Mean time to death and mean time to clinical resolution**

Serotypes	Death/re-solution	O1	O2	O6	O10	O11	PL5	NT
**Time to death in days, mean** ± **SD**	**(n = 20)**	(n = 4)	(n = 0)	(n = 6)	(n = 3)	(n = 3)	(n = 3)	(n = 1)
	**11.4 ± 7.4**	12.0 ± 8.1	NA	10.2 ± 7.1	16.0 ± 7.2	12.3 ± 4.2	8.3 ± 4.0	7
**Time to clinical resolution in days, mean** ± **SD**	**(n = 75)**	(n = 4)	(n = 10)	(n = 28)	(n = 7)	(n = 8)	(n = 12)	(n = 6)
	**12.1 ± 5.9**	11.0 ± 5.8	9.9 ± 4.0	11.6 ± 6.1	13.1 ± 4.3	13.4 ± 7.2	13.3 ± 5.9	10.8 ± 4.1

NT, non typeable; PL5, sum of all serotypes with a prevalence of less than 5%; SD, standard deviation.

## Discussion

Our study shows that, of the 20 different serotypes of *P. aeruginosa*, O6 (29%) and O11 (23%) were the most prevalent serotypes, responsible for the majority of nosocomial pneumonia. Across all serotypes, clinical outcome correlates strongly to APACHE score. Mortality and clinical resolution tend to be worse in patients infected by *P. aeruginosa* serotype O1 and better in patients infected by serotypes O2 and NT and O6.

### Prevalence of *Pseudomonas aeruginosa*serotypes

While the prevalence of *P. aeruginosa* serotype varies from one hospital to another and from one country to another, O6 and O11 are often the most prevalent serotypes reported in previous studies [10, 14, 25–30]. It should be pointed out that the prevalence of *P. aeruginosa* serotypes reported in previous studies was obtained either from multiple infectious sites or from a single hospital or country. To the best of our knowledge, our study is the first assessing the prevalence of *P. aeruginosa* serotypes in nosocomial pneumonia from different hospitals of different countries.

Although the prevalence of serotypes is different among the countries and investigator sites (Figure 2 and Table 3), the overall rates of prevalence of the most common *P. aeruginosa* serotypes observed in patients with nosocomial pneumonia were O6 and O11. After exclusion of the largest enrollment site from the analysis, O6 and O11 remain the most prevalent serotypes, suggesting that investigator site effect does not influence interpretation of the finding. This result is comparable with those previously reported in the literature [10, 15, 25, 26, 28]. In approximately 8% of cases, the standard method failed to detect serotype because of self-agglutinating or non-agglutinating strains and the samples were classified as “not typeable”. This proportion is less than those previously reported [11, 18, 27].

### Clinical outcome and *Pseudomonas aeruginosa*serotypes

Age and APACHE II score were found to be the predictive risk factors for nosocomial pneumonia caused by *P. aeruginosa*. This result indicates that mortality and clinical and microbiological resolutions were strongly correlated with severity of the patients at the initial phase of pneumonia [31–33].

The relationship between virulence of *P. aeruginosa* and different serotypes has been investigated in a few studies [12–14]. It has been shown that clinical strains lacking B-band O antigen increase TTSS and virulence [34]. On the other hand, it was found that strains secreting ExoU were frequently serotyped as O11 [12, 15]. ExoU is one of the toxins secreted by TTSS and contributes to epithelial cell toxicity, lung injury, and sepsis in infected animals [35, 36]. Exo S and Exo T disrupt the host cell actin cytoskeleton, block phagocytosis, and are associated with mortality in animal models [37]. Furthermore, TTSS is associated with persistent VAP caused by *P. aeruginosa* and poor clinical outcome [38, 39]. Faure et al. reported that, among 13 *P. aeruginosa* O1 strains, 7 secreted Exo S [12]. Recently, Le Berre et al. showed that the O11 serotype, elastase production, and TTSS were associated with increased lung injury in a murine model of pneumonia [15]. They found that serotype O11 strains were significantly more virulent than non-typeable strains and serotype O6; O11 strains were associated with a positive (ExoU) phenotype, whereas O6 strains were associated with negative ExoU phenotype [12, 15]. These results suggest that clinical outcome of patients with pneumonia caused by *P. aeruginosa* could be related to serotypes.

This article reports on the first ever study to assess the clinical outcome of nosocomial pneumonia caused by a range of different serotypes of *P. aeruginosa* in critically ill patients under standard ICU management. Interestingly, the results show that the mortality rates for serotypes O1, O10, and O11 were in line with the expected mortality rates estimated by the severity of the patients (APACHE II score). Infections with serotypes O6, O2, and NT serotypes, however, had lower mortality than expected. There was a trend toward poorer outcome of patients with pneumonia caused by serotypes O1.

By cross-referencing prevalence and mortality rates, one can see that, while the O6 serotype is the most prevalent (29%), it has lower levels of continuation (8%) and death (17%) and higher resolution rates (75%) than the second most prevalent serotype O11 (prevalence 23%, continuation 21%, death rate 21%, and resolution 57%). This finding could be explained by the absence of ExoU with serotype O6 and presence of ExoU with serotype O11 as previously reported [15]. Consequently, compared with O6, O11 may be a more clinically relevant target for therapy in terms of patient outcomes [19]. Further study with larger groups is required in order to draw firm conclusions.

### Methodological limitations

First, the prevalence obtained from our study may not be applied globally, because of an absence of data from additional countries known for their high prevalence of pneumonia caused by *P. aeruginosa*, such as Spain, Italy, Greece, the US, Mexico, Canada, and Japan. Serotypes may also vary from hospital to hospital within a given country and within the same hospital when assessed at different times. The most prevalent serotypes obtained in our study are, however, comparable to those previously reported in the literature. Second, the potential relationship between serotypes, virulence factors, and antibiotic resistance was not studied. Patients hospitalized in the same ICU could have a *P. aeruginosa* clone with the same virulence. Moreover, *P. aeruginosa* strains with the same serotype could be different clones exhibiting different virulence. Third, interpreting the results on clinical outcome is limited due to the small size of the individual serotype groups. However, these preliminary data provide relevant information for further investigations.

Further larger clinical study combining clinical outcome with distribution of virulence factors by serotypes and identification of specific clones by pulsed-field gel electrophoresis is needed to enhance the implication of these clinical findings [40].

## Conclusions

O6 and O11 are the most prevalent serotypes in nosocomial pneumonia caused by *P. aeruginosa*. Mortality tends to be worse with O1 and better with O2; clinical resolution tends to be better with O2 and O6 compared with other serotypes, but it is difficult to draw firm conclusions given the small number of strains in each serotype group. Further large-powered multicenter study is required to assess clinical outcome of nosocomial pneumonia caused by *P. aerginosa* serotypes, particularly O6 and O11, the two serotypes most frequently encountered in critically ill patients.

## Key messages

- ● In *Pseudomonas aeruginosa* nosocomial pneumonia, the most prevalent serotypes are O6 and O11.
- ● Across all serotypes, mortality and clinical resolution of pneumonia correlate strongly to APACHE score.
- ● Clinical outcome tends to be worse with serotype O1 and better with O2, but a firm conclusion needs a larger group size.

## Abbreviations

APACHE II: Acute Physiology and Chronic Health Evaluation II
BAL: bronchoalveolar lavage
CFU: colony-forming units
CPIS: clinical pulmonary infection score
EOS: end of study
ETA: endotracheal aspirate
ExoU: exotoxin U
HAP: hospital-acquired pneumonia
IATS: international antigenic scheme
ICU: intensive care unit
LPS: lipopolysaccharide
MCPIS: modified clinical pulmonary infection score
mini-BAL: protected mini-bronchoalveolar lavage
NT: not typeable
PL5: sum of all serotypes with a prevalence of less than 5%
SOFA: sequential organ failure assessment
TTSS: type III secretion system
VAP: ventilator-associated pneumonia.
